# Identification of a *Rice stripe necrosis virus *resistance locus and yield component QTLs using *Oryza sativa *× *O. glaberrima *introgression lines

**DOI:** 10.1186/1471-2229-10-6

**Published:** 2010-01-08

**Authors:** Andrés Gonzalo Gutiérrez, Silvio James Carabalí, Olga Ximena Giraldo, César Pompilio Martínez, Fernando Correa, Gustavo Prado, Joe Tohme, Mathias Lorieux

**Affiliations:** 1 Agrobiodiversity and Biotechnology Project, International Center for Tropical Agriculture (CIAT), A.A. 6713, Cali, Colombia; 2 Institut de Recherche pour le Développement (IRD), Plant Genome and Development Laboratory, UMR 5096 IRD-CNRS-Perpignan University, 911 Av. Agropolis, 34394 Montpellier Cedex 5, France. Current address: Agrobiodiversity and Biotechnology Project, CIAT, A.A. 6713, Cali, Colombia; 3 Agrobiodiversity and Biotechnology Project, International Center for Tropical Agriculture (CIAT), A.A. 6713, Cali, Colombia. Current Address: RiceTec, Inc., PO Box 1305, Alvin, Texas 77512, USA

## Abstract

**Background:**

Developing new population types based on interspecific introgressions has been suggested by several authors to facilitate the discovery of novel allelic sources for traits of agronomic importance. Chromosome segment substitution lines from interspecific crosses represent a powerful and useful genetic resource for QTL detection and breeding programs.

**Results:**

We built a set of 64 chromosome segment substitution lines carrying contiguous chromosomal segments of African rice *Oryza glaberrima *MG12 (acc. IRGC103544) in the genetic background of *Oryza sativa ssp. *tropical *japonica *(cv. Caiapó). Well-distributed simple-sequence repeats markers were used to characterize the introgression events. Average size of the substituted chromosomal segments in the substitution lines was about 10 cM and covered the whole donor genome, except for small regions on chromosome 2 and 4. Proportions of recurrent and donor genome in the substitution lines were 87.59% and 7.64%, respectively. The remaining 4.78% corresponded to heterozygotes and missing data. Strong segregation distortion was found on chromosomes 3 and 6, indicating the presence of interspecific sterility genes. To illustrate the advantages and the power of quantitative trait loci (QTL) detection using substitution lines, a QTL detection was performed for scored traits. Transgressive segregation was observed for several traits measured in the population. Fourteen QTLs for plant height, tiller number per plant, panicle length, sterility percentage, 1000-grain weight and grain yield were located on chromosomes 1, 3, 4, 6 and 9. Furthermore, a highly significant QTL controlling resistance to the *Rice stripe necrosis virus *was located between SSR markers RM202-RM26406 (44.5-44.8 cM) on chromosome 11.

**Conclusions:**

Development and phenotyping of CSSL libraries with entire genome coverage represents a useful strategy for QTL discovery. Mapping of the RSNV locus represents the first identification of a genetic factor underlying resistance to this virus. This population is a powerful breeding tool. It also helps in overcoming hybrid sterility barriers between species of rice.

## Background

Asian rice (*Oryza sativa *L.) is one of the most important food crops for mankind and is considered to be a model system for molecular genetic research in monocots, due to its small genome size and its synteny with other cereal crops [1,2]. Recent advances in large-scale genomic research has provided extremely useful tools, such as a complete, high-quality genome sequence [3], Bacterial Artificial Chromosome libraries [4], insertional mutant collections [5], and the discovery of new molecular markers [6-8]. Plant breeders and geneticists have taken advantage of these advances by using both cultivated and wild germplasm as new sources of genetic variation to facilitate identification of genes and QTLs of economic importance, contributing to an increased rice production.

Although methodologies for mapping genes or QTLs underlying quantitative traits have made considerable progress, the need to develop new population types to facilitate the study of alleles from wild species, has been pointed out. These materials would allow identification and use of new sources of allelic variation that have not been sufficiently exploited yet [9-14]. Different types of segregating populations, like Recombinant Inbred Lines (RIL), Doubled Haploids (DH), Backcross (BC) or F_2_/F_3 _populations have been extensively used for QTL mapping. Nevertheless, these populations do not have sufficient power in detecting QTLs with minor effects, at least when standard population sizes of a few hundreds of segregating individuals are used [11,15]. Moreover, in the case of interspecific crosses, hybrid sterility often hampers developing such population types. To circumvent these issues, researchers have developed novel population types, which are all very similar in essence: Introgression Lines (ILs) in tomato [11]*Brassica napus *[16] and *Brassica oleracea *[17], Stepped Aligned Inbred Recombinant Strains (STAIRS) in *Arabidopsis *[15], Recombinant Chromosome Substitution Lines (RCSL) in barley [18], introgression libraries in rye [19], Chromosome Segment Substitution Lines (CSSL) or Single Segment Substitution Lines (SSSL) in rice [9,20-31]. In these populations, which all belong to the generic introgression lines family, the iterative backcrossing process often makes it possible to recover a partial or complete fertility of the progeny.

Libraries of introgression lines are produced by successive backcrossing (generally three to four generations) to the recurrent parent. The introgressed fragments can be monitored using molecular markers, either in each generation or at chosen stages. Fixation of the materials is obtained by either selfing or using the double-haploid methodology (e.g. by anther culture). As a result, each line possesses one or few homozygous chromosomal fragments of the donor genotype, introgressed into a recurrent background genome. These fragments should be arranged contiguously from the first to the last chromosome, either manually or using a computer software-aided process (graphical genotyping). The whole donor genome is thus represented by a set of small, contiguous overlapping fragments.

The objective of this paper is to describe the development and selection of a CSSL library derived from an interspecific cross between *O. sativa *L. and *O. glaberrima *Steud., the cultivated African rice species. In order to illustrate the usefulness of this resource for genetic analyses and breeding purposes, we present a QTL detection analysis for grain yield, yield components and resistance to *Rice stripe necrosis virus *(RSNV).

## Results

### Description of the CSSL population

**Figure 1:**
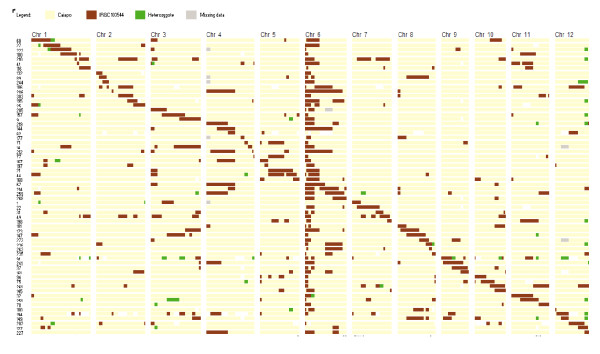
**Graphical representation of the genotypes of 64 BC3DH lines selected from a library of 312 lines**. The 12 rice chromosomes are displayed vertically. They are covered by 125 evenly dispersed SSR markers. The genotypes are displayed horizontally. Color legend indicates the allelic status of chromosomes, where "Recurrent" means homozygous for the Caíapo allele and "Donor" means homozygous for the MG12 allele.

The CSSL Finder program selected a subset of 125 SSR markers properly distributed across the twelve rice chromosomes. On this basis, searching for CSSL candidates led to a set of sixty-four lines (Figure 1). Average size of the substituted chromosomal segments in the CSSLs was of 10 cM and covered the whole *O. glaberrima *genome, except for small regions landmarked by markers RM71-RM300 (43.8-65.9 cM) on chromosome 2 and RM185-RM241 (93.8-135.0 cM) on chromosome 4. The proportions of Caiapó and MG12 in the CSSL lines were 87.59% and 7.64%, respectively. The remaining 4.78% corresponded to heterozygotes and missing data. The number of introgressed segments varied between 2 to 8 per line. We observed several lines with a few heterozygous chromosomal regions, for which pollen contamination that occurred in the field between lines of the population is the most probable explanation. Additional backcrossing (2-3) with marker-assisted monitoring is currently carried out to purify the genetic background of the 64 lines.

### Trait correlations

Correlation coefficients among yield and yield component traits were tested for significance at *P *< 0.05 and *P *< 0.01, and are presented in Table 1. Coefficients of phenotypic correlation were low, indicating the complexity of relationships between these traits. Positively correlated traits (*P *< 0.01) were plant height with yield (R^2 ^= 0.376) and panicle length (R^2 ^= 0.548), and sterility percentage with tiller number per plant (R^2 ^= 0.295). The observed correlation between plant height and yield corroborates previous yield-associated QTL studies in rice [32,33]. Panicle length is largely proportional to plant height, explaining the relatively high R^2 ^value. Negatively correlated traits (*P *< 0.01) were plant height with 1000-grain weight (R^2 ^= - 0.172), and, as expected, sterility percentage with yield (R^2 ^= - 0.244).

**Table 1:** Correlation coefficients (R^2^) between yield and yield component traits in Caiapo × MG12 interspecific cross

*Traits*	*Plant height*	*Tillering*	*Yield*	*Panicle Length*	*Sterility*
*Tillering*	0.079				
*Yield*	0.376 **	0.015			
*Panicle Length*	0.548 **	-0.070	0.110		
*Sterility*	0.131 *	0.295 **	-0.244 **	0.119 *	
*1000-grain weight*	-0.172 **	-0.118 *	-0.140 *	-0.056	-0.084

Units: Plant height (cm), Tillering (tiller number per plant), Yield (Kg/Ha), Panicle length (cm), Sterility percentage (number of empty spikelets/total number of spikelets), and 1000-grain weight (grams)

* P < 0.05

** P < 0.01

### QTL analysis for yield and yield components

**Figure 2:**
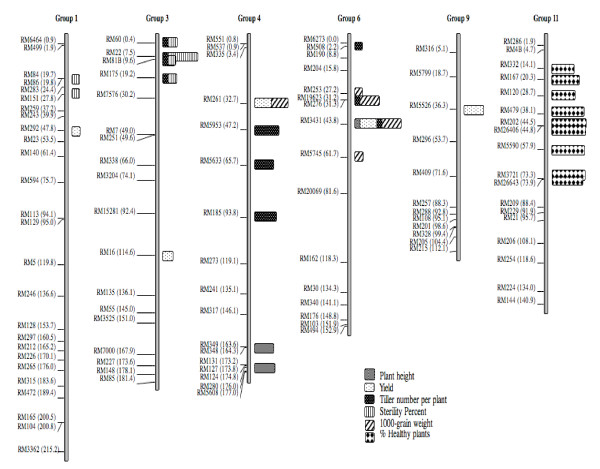
**Genetic locations of the 15 QTLs for yield components an RSNV resistance (% Healthy plants) detected in this work**. On the left, SSR marker positions and distances (cM) based on IR64/TOG5681 genetic linkage map, developed at CIAT in 2007 (our unpublished data). On the right, QTL for yield, yield components and RSNV resistance on chromosomes 1, 3, 4, 6, 9 and 11.

**Figure 3:**
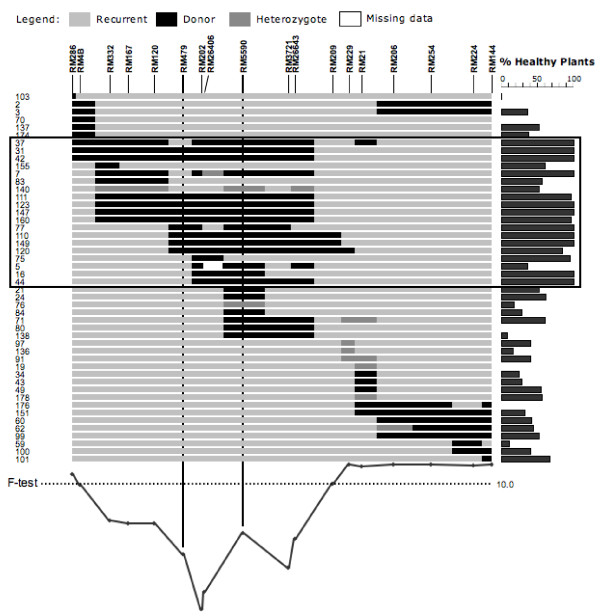
**Major QTL for *O. glaberrima *Acc. MG12 resistance to the Rice Stripe Necrosis Virus (RSNV), located on rice chromosome 11 between SSR markers RM479 and RM5590 (*F *= 70.63, *P *~ 0.0)**. On the right, solid grey bars indicate the value of percentage of healthy plants for each line. The resistant lines (% of healthy plants > 85) are located within the black frame. The most probable location of the resistance QTL is given by the intersection of the black frame and the positions of the markers RM479 and RM5590, which define a common introgressed region between the resistant lines.

Fourteen QTLs were found for plant height (*PTHT)*, yield (*YLD*), tiller number per plant (*TINB*), 1000-grain weight (*TGRWT*) and sterility percentage (*ST*) located on chromosomes 1, 3, 4, 6 and 9. A major QTL for RSNV was detected on chromosome 11 (Table 2; Figures 2, 3). All QTLs were detected by both the single-marker ANOVA1 and interval mapping-based methods (IM and CIM), indicating their robustness for QTL detection for this type of populations.

**Table 2:** QTLs detected for five yield and yield components traits and RSNV resistance in MG12 × Caiapó BC3DH population

*Traits*	*QTL*	*Linkage group*	*Peak Marker*	*^a^Position*	*LOD*	*^b^R^2^*	*F*
Plant height	PTHT-4	4	RM124	174.8	6.7	7.0	17.34
	PTHT-6	6	RM3431	43.8	12.9	16.0	34.7
Tiller number per plant	TINB-3	3	RM60	0.4	6.2	7.0	24.22
	TINB-4	4	RM5953	47.2	4.9	6.5	25.03
	TINB-6	6	RM3431	43.8	3.3	4.8	30.40
Yield	YLD-1	1	RM292	47.8	3.8	4.0	16.60
	YLD-3	3	RM16	114.6	10.5	14.0	20.08
	YLD-4	4	RM261	32.7	3.8	5.0	15.40
	YLD-6	6	RM3431	43.8	7.2	11.0	25.63
	YLD-9	9	RM5526	36.3	2.8	3.0	16.10
Sterility Percent	ST-1	1	RM86	19.8	2.8	17.0	15.99
	ST-3	3	RM22	7.5	7.8	10.0	31.14
1000-grain weight	TGRWT-4	4	RM261	32.7	5.0	8.0	32.69
	TGRWT-6	6	RM3431	43.8	7.8	11.0	39.49
RSNV	RSNV-11	11	RM202	44.5	16.0	32.0	70.62

^a^Position: Absolute position on the chromosome, indicated in cM (centimorgans)

^b^R^2 ^: Percentage of phenotypic variation explained by the QTL

#### Plant height (PTHT)

Two QTLs (*PTHT*-*4 *and *PTHT*-*6*) with a maximum *F*-test value of 17.34 and 34.7, respectively were detected on chromosomes 4 and 6. These QTLs were also reported by [34] in the same population, but based on phenotypic evaluation in a different environment.

#### Tiller number per plant (TINB)

For this trait, three QTLs (*TINB*-*3*, *TINB*-*4 *and *TINB*-*6*) on chromosomes 3, 4 and 6 were detected with a maximum *F*-test value of 24.22, 25.03 and 30.40, respectively. On a region near *TINB*-*4*, RM185 on chromosome 4 was reported as marking a QTL for tiller number in the IR64/Azucena DH population developed at the International Rice Research Institute (IRRI) [35]http://www.gramene.org.

#### Yield (YLD)

Five QTLs (*YLD-1, YLD-3, YLD-4*, *YLD-6 *and *YLD*-9) were located on chromosomes 1, 3, 4, 6 and 9 with a maximum *F*-test value: 16.60, 20.08, 15.40, 25.63 and 16.10, respectively. One QTL was reported for yield in a region of approximately 2 cM on chromosome 1, near QTL *YLD-1 *[34]. A QTL on chromosome 3 near the *YLD-3 *position was identified by [36] in the Nipponbare/Kasalath F_2 _population.

#### Sterility percentage (ST)

Two QTLs (*ST-1 *and *ST-3*) were mapped on chromosomes 1 and 3 with a maximum *F*-test value of 15.99 and 31.14, respectively. A QTL was reported for spikelet sterility within the interval 16.40-27.80 cM on chromosome 1 [37], near QTL *ST-1 *(19.0 cM) reported in this study. A QTL was reported in the region of *ST-3 *for pollen fertility in the cross Taichung 65/*O. glaberrima *[38].

#### 1000-grain weight (TGRWT)

Two QTLs (*TGRWT-4 *and *TGRWT-6*) were detected on chromosomes 4 and 6 with maximum *F*-test value of 32.69 and 39.49, respectively [39] reported a QTL for 100-grains weight on RM261 locus marker, at the same locus as *TGRWT-4.*

### QTL analysis for resistance to RSNV

Using both CSSL Finder and WinQTLCart software, one highly significant QTL with an *F *= 64.40 could be located on chromosome 11. The QTL region was saturated with downstream and upstream SSR markers delimiting this QTL (Figures 2 and 3). Analysing the recombination events in the region allowed us to semi-fine map the RSNV major QTL, between SSR markers RM202-RM26406 (44.5-44.8 cM).

## Discussion

### Segregation distortion

The phenomenon of segregation distortion (SD), defined as a deviation from the expected Mendelian segregation ratios in a segregating population, has been reported in several crops. In rice, this effect is often due to sterility genes located on several chromosomal regions. Genetic interactions, genes with variable effects in regeneration by anther culture and physiological and/or environmental factors can also lead to SD [40]. 37% (74) of the markers showed distortion in favour of MG12 alleles on chromosomes 1, 2, 3 and 6. As expected, the strongest segregation distortion was found at the short arm of chromosome 6, at markers RM6273 and RM204 (0.0-15.8 cM) [41-43]. This region corresponds to the genomic location of the *S_1 _*locus, a sporo-gametophytic sterility factor identified in previous studies. The other distorted regions matched with the chromosomal locations of *O. sativa *× *O. glaberrima *sterility loci described so far: *S_33(t) _*on chromosome 1 [44], *S_29(t) _*on chromosome 2 [45], *S_19 _*and *S_34(t) _*on chromosome 3 [46,47].

### Comments on QTLs for yield components

**Figure 4:**
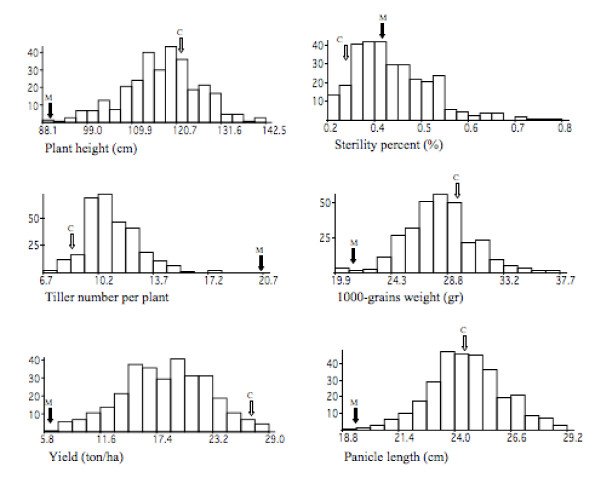
**Frequency distribution of yield component traits in 312 BC_3_F_1_DH lines**. Parental values are indicated by arrows. C = Caíapo (*O. sativa*), M = MG12 (*O. glaberrima*).

Yield is a complex trait controlled by many genes of major or minor effect [32]. QTLs for yield found in the present study were associated with small effects that are co-localized with QTLs of the group of M-QTLs (main-effect QTLs) identified in other studies. M-QTLs represent more than 90% of the QTLs reported to date [48]. Also, transgressive segregation was observed for all traits except tillering (Figure 4), demonstrating that interspecific crossing enhanced the possibility of introgressing genetic variability in cultivated rice [49,50]. Although several QTLs were detected on the short arm of chromosome 6, they should be carefully considered, because their effects could have been overestimated due to the strong segregation distortion affecting this region.

### QTLs for RSNV resistance

To our knowledge, this is the first identification of a genetic factor underlying resistance to the RSNV disease. In order to better elucidate the bases of genetic control of RSNV resistance, fine mapping of this region is being envisaged using recombinant event analysis in the BC_4_F_2_/F_3 _lines that we produced in 2007.

### Efficiency of CSSL lines for rice breeding

Breeding strategies such as marker-assisted selection (MAS) or marker-assisted backcrossing (MAB) require comprehensive dissection and understanding of the complex traits measured. Development of genetics resources such as CSSL lines will greatly facilitate the detection of naturally occurring allelic variation in rice and will help to acquire a better knowledge of target traits [9,12,13,51]. Phenotyping strategies based on CSSL populations present the advantage of a relatively small number of lines to evaluate, with the possibility of replicating evaluations over space and time. This should lead to better quality data in the case of complex, time-consuming or expensive phenotypic evaluations. Genetic dissection of complex traits by associating genetic variation with introgressed fragments allows us to reduce interference effects between QTLs. This helps to understand the genetic bases of reproductive barriers between species, and provides a powerful approach for QTL identification, fine mapping of QTLs, laying the bases for both marker-assisted selection and map-based cloning strategies based on exploitation of wild alleles. Comparison of phenotypic values between any line of the population and the recurrent parent generates high statistical power. CSSL lines can be crossed in different ways in order to study epistatic interactions between QTLs, develop Near-Isogenic Lines (NIL) and do QTL pyramiding [16,26,31,52].

## Conclusion

### Usefulness of CSSL libraries

Wild and cultivated African rice species have been shown to be valuable sources of alleles associated with traits of agronomic importance [12,43]. However, they carry many undesirable alleles that may show strong linkage to favorable alleles, linkages that usually are very difficult to break up by conventional crossing. CSSL lines give access to the original exotic allelic source, providing an elegant way of circumventing this issue, thus representing a useful and powerful tool for genetics and breeding approaches. They constitute a very useful genetic resource for studying both inheritance of agronomically important traits and directing their incorporation as progenitors in breeding programs for the development of elite germplasm with exotic characteristics of interest. The set of CSSL lines presented in this study is available to the rice community through both the CIAT Rice Outcome Product Line and the Generation Challenge Programme. Several research teams around the world are already using this population in their effort to locate, map and utilize new alleles associated with traits of economic importance.

### Development of new CSSL libraries with wild genomes

The genetic diversity of crop plants has been narrowed down due to the domestication process and decades of selection. Exotic genetic resources such as wild rice species can be successfully exploited to increase allelic variability into elite lines [53,54]. Within the framework of a Generation Challenge Programme project, we are now developing a series of new CSSL populations, using wild AA-genome rice species (*O. rufipogon, O. glumaepatula, O. meridionalis and O. barthii*) as donors. Associated partners to this effort are EMBRAPA-CNPAF (Brazil), WARDA (Benin) and Cornell University (USA). These wild species as well as African cultivated rice show adaptation to biotic and abiotic constraints associated with specific geographic regions. Transgressive segregation has been demonstrated in several studies [49,55]. The development of libraries of introgression lines makes immediate use possible for plant breeders and will simultaneously serve to enhance our understanding of the wild/cultivated allelic genetic interactions. We hope that the results of this work will contribute to a better understanding of plant performance key components and to the development of new improved rice cultivars.

## Methods

### Plant materials

The recurrent parent Caiapó (*O. sativa ssp. tropical japonica*) is a commercial rice variety developed by EMBRAPA-CNPAF (Goiania, Brazil) and has been cultivated since 1992 in Brazil and other places in Latin America and the Caribbean. This variety is characterized by presenting yields of 2.5 tons/ha under upland conditions, long grain type, medium growth cycle, tolerance to leaf blast (*Magnaporthe grisea*), moderate resistance to neck blast and tolerance to aluminium toxicity, acid soil conditions and drought [56]. The donor parent MG12 (acc. IRGC103544) is an accession of the African cultivated rice species, *O. glaberrima*. This species is grown in West Africa and shows several negative characteristics with respect to the Asian *O. sativa*, like shattering, brittle grain and poor milling quality. More importantly, it consistently shows lower yields than *O. sativa*. However, African rice often shows more tolerance to fluctuations in water depth, iron toxicity, infertile soils, severe climatic conditions and human neglect, and exhibits better resistance to various pests and diseases like nematodes (*Heterodera sacchari *and *Meloidogyne sp*.), African gall midge, RSNV and *Rice yellow mottle virus *(RYMV) [57-61].

### Population development

**Figure 5:**
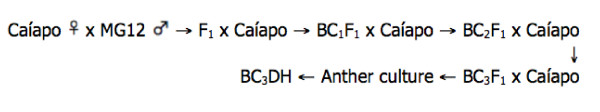
**Development scheme of the population of BC3DH lines derived from Caíapo (*O. sativa*) × MG12 (*O. glaberrima*) interspecific cross**.

The population was developed at the International Center for Tropical Agriculture (CIAT) headquarters, in Cali, Colombia, starting in 1997. The scheme applied for population development is shown in Figure 5. Accession MG12 was used as the male parent of the F_1 _hybrid. F_1 _plants were completely androsterile and 20 individuals were randomly selected as females for backcrossing with the recurrent parent Caiapó. A total of 154 BC_1_F_1 _plants were produced and then successively backcrossed to Caiapó until the BC_3_F_1 _generation. Anthers were collected from the BC_3_F_1 _plants and processed through *in vitro *culture to generate double haploids (DH) as described by [62]. As a result, 695 BC_3_F_1_DH lines were obtained and multiplied for seed under irrigated field conditions in 2000. Subsequently, a subset of 312 BC_3_F_1_DH lines offering a good representation of the observed phenotypic variability was selected as a mapping population for agronomic evaluation and molecular characterization [Additional file 1: Figure S1].

### Phenotypic evaluation

The mapping population and the parent accessions (as controls) were first evaluated in replicated field plots in Colombia at CIAT headquarters in 2001. Materials were planted under irrigated conditions in a randomized complete block design arranged in two rows, where each row was 5 m long with a spacing of 30 × 30 cm (20 plants/row), with three replications. Transplanting was done at twenty-five days after sowing. Five plants per BC_3_F_1_DH line were randomly selected and then evaluated for six agronomic traits: plant height (PTHT), tiller number (TINB), panicle length (PNLG), percentage of sterility (ST), 1000-grain weight (TGRWT) and grain yield (YLD). A second field experiment with the BC_3_F_1_DH lines and the two parents was planted in a randomized complete block design with two replications at the Rice Research Station, Crowley, Louisiana [34] in 2002.

**Figure 6:**
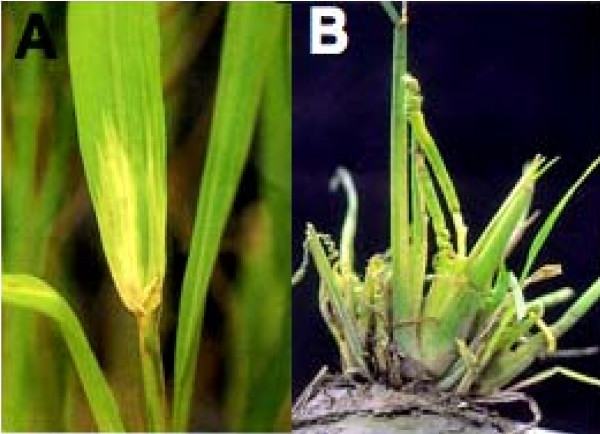
**Characteristic symptoms of the disease "crinkling" caused for RSNV in rice plants (A) Yellow stripes on leaves or foliar striping and (B) Crinkling or deformation (Courtesy: Gustavo Prado, Rice Pathology Laboratory, CIAT, Cali, Colombia)**.

*Rice stripe necrosis virus *is a furovirus associated with the disease known as crinkling, hence its common name, "*crinkle virus*". It was first reported in West Africa in the late 1970s [63]. Later on, in 1991, the virus was found in South America, in the Colombian Department of Meta and was locally called "*entorchamiento*" [64]. Symptoms include seedling death, foliar striping and severe plant malformation. This disease can provoke yield losses of up to 40% in highly infected fields. Since *O. glaberrima *was shown to be highly resistant to RSNV [60], we took advantage of the usefulness and potential of the CSSL lines to search for QTLs for RSNV resistance. In order to screen the lines for their resistance to RSNV, infested soil from farmer's field was used as inoculum. The level of soil infestation was tested by planting the highly susceptible rice cultivar Oryzica 3 in several pots containing the infested soil. The infested soil was used if the incidence of RSNV infected plants on the susceptible check was above 80%. The virus incidence on the mapping population was evaluated in 178 lines by counting the number of plants showing the characteristic symptoms of the disease, including: 1) crinkling or deformation, 2) yellow stripes on leaves or foliar striping, 3) stunting of plants (Figure 6) and 4) dead plants. Number of healthy plants was also recorded. The highly susceptible cultivar Oryzica 3 was used in each experiment as a control and indication of the disease pressure. Ten plants per line were evaluated. Lines with a percentage of healthy plants superior to 85% were considered as resistant, while the other ones were considered as susceptible. These evaluations were carried out in the greenhouse of the CIAT's Rice Pathology Laboratory, where the average of both relative humidity was 80 percent and the temperature 25°C. A randomized complete block design with four replications with ten plants per pot was used. The experiment was replicated two times over a period of six months with a total of 80 plants evaluated for each genotype. Two evaluations were made, the first one 30 days after planting and the second one 60 days after planting. Final line reaction was based on the second evaluation. In each experiment the plants were fertilized with a commercial dose of Nitrogen equivalent to 200 KgN/ha in order to favour the development and high incidence of the disease.

### DNA marker analysis

Total DNA was extracted from frozen leaf tissue based on a slightly modified version of the Dellaporta protocol (our unpublished data). Subsequently, quality and quantity of DNA was evaluated on 0.8% agarose gel stained with ethidium bromide. A total of 200 polymorphic simple sequence repeats (SSR) loci distributed across the twelve rice chromosomes with an average spacing of 8.0 cM was used. Most of these SSR markers were selected from the Universal Core Genetic Map (UCGM) of rice developed at CIAT Rice Genetics and Genomics group [65]. The UCGM was developed from the list of 18,000 SSRs published in IRGSP (2005). Polymerase chain reactions (PCR) were performed in a total volume of 15 *μ*L containing 20 ng/μL of DNA template, 1X PCR buffer, 2.5 mM of MgCl_2 _(or 1.5 to 2.0 mM for some specific pairs of primers), 0.2 mM of d-NTP, 0.13 *μ*M of each primer and 1 U/*μ*L *Taq *DNA polymerase. Amplification was run on MJ Research PTC-225 (384 well) thermocycler with the following program: 94°C for 3 min; 29 cycles at 94°C for 30 s, 55°C for 45 s (modified for some specific pairs of primer), 72°C for 1 min; 72°C for 5 min. PCR products were separated on 4% high-throughput agarose gel for markers that showed a polymorphism size higher than 10 bp, and stained with ethidium bromide. For polymorphism lower than 10 bp, PCR products were separated using 6% denaturing polyacrylamide gel followed by silver staining, as described in the Promega Technical Manual [66].

### Selection of a subset of CSSLs

Selection of a subset of introgression lines that cover the entire donor genome was carried out with the help of the CSSL Finder v. 0.84 computer program [67]. CSSL Finder was designed to search for a subset of CSSL that optimizes specific parameters: target size of introgression segments, percentage of donor genome and number of introgressed fragments. It also makes it possible to define the minimum set of lines that cover the entire donor genome, according to the same parameters. Subsequently, graphical genotypes of the candidate lines can be displayed. CSSL Finder is available at no cost at http://mapdisto.free.fr.

### Statistical analyses

As the coordinates of SSR markers of the UCGM are physical positions on the rice pseudomolecules, it was necessary to convert them to centimorgans (cM) in order to obtain QTL confidence intervals comparable to those obtained in other studies. For this purpose, we used a genetic linkage map obtained from a BC_1_F_1 _population derived from the cross IR64 (*O. sativa ssp. indica*) × TOG5681 (*O. glaberrima*) (our unpublished data). The map was constructed using the computer program MapDisto v. 1.7 [68]http://mapdisto.free.fr. For each marker, a chi-squared test (P < 0.01) was performed to identify markers with segregation distortion. Correlation between the traits evaluated was calculated using the QGene v. 3.07 program [69], and tested using significance levels of 0.05 and 0.01. As several introgression events are present at each marker position in the complete set of 312 lines, we used standard methods to identify QTLs linked to the segregating traits. A QTL analysis for the evaluated traits was done using both the CSSL Finder v. 0.84 and the MapDisto v. 1.7 programs, which basically perform a single-marker ANOVA1 *F*-test. We considered the *F*-test as significant when its value was higher than 15. CSSL Finder was used to display graphical genotyping of subsets of fifteen lines that presented the most extreme phenotypic value for each trait, in order to confirm each detected QTL. Interval mapping (IM) and composite interval mapping (CIM) analyses using WinQTLCart v. 2.5 [70] were also performed. Significant QTLs found using *F*-test, IM and CIM methods were compared with previous studies.

## Authors' contributions

AGG and OXG carried out the QTL analyses and molecular marker studies, SJC developed the interspecific population and carried out the field testings (yield components), CPM conceived and leaded field testings (yield components), FC and GP conducted greenhouse RSNV evaluations, JT and CPM conceived the design of the population, ML developed the methodology to identify the CSSL and coordinated the statistical analysis. AG drafted the manuscript. ML and CPM revised the manuscript. All authors read and approved the final manuscript.

## Supplementary Material

Additional file 1 **Figure S1. The Caiapó × IRGC103544 (MG12) population of interspecific introgressed lines**. General view of the Caiapó × IRGC103544 (MG12) population of BC_3_F_1_DH lines in the field. Click here for file
