# Supplementary figures and images for: Identification of a Rice stripe necrosis virus resistance locus and yield component QTLs using Oryza sativa × O. glaberrima introgression lines
Source: BMC Plant Biol. 2010 Jan 8;10:6. doi: 10.1186/1471-2229-10-6 (PMC2824796; doi:10.1186/1471-2229-10-6)

**
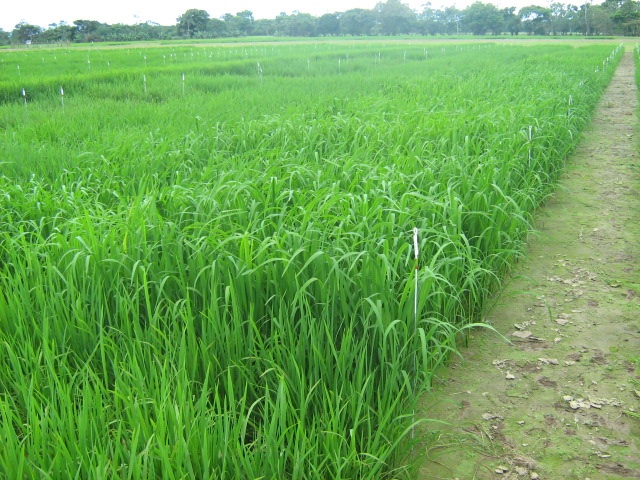
**

Supplement: Additional file 1 — Figure S1. The Caiapó × IRGC103544 (MG12) population of interspecific introgressed lines. General view of the Caiapó × IRGC103544 (MG12) population of BC3F1DH lines in the field. [file 1471-2229-10-6-S1.DOC]
